# Update and New Implementation of the MIRAGE Reporting Guidelines for Mass Spectrometry Experiments in Glycoscience

**DOI:** 10.1016/j.mcpro.2025.101473

**Published:** 2025-11-24

**Authors:** Frederique Lisacek, William E. Hackett, Morten Thaysen-Andersen, Niclas G. Karlsson, Joshua Klein, Daniel Kolarich, Marissa L. Maciej-Hulme, Shujiro Okuda, Nicolle H. Packer, Weston B. Struwe, Yushi Takahashi, Michael Tiemeyer, Joseph Zaia, Kiyoko F. Aoki-Kinoshita, Carsten Kettner

**Affiliations:** 1 Proteome Informatics, SIB Swiss Institute of Bioinformatics, Geneva, Switzerland; 2 Computer Science Department, University of Geneva, Geneva, Switzerland; 3 Program for Bioinformatics, Boston University, Boston, Massachusetts, USA; 4 Centre of Excellence in Synthetic Biology, School of Natural Sciences, Macquarie University, Sydney, Australia; 5 Institute for Glyco-core Research (iGCORE), Nagoya University, Nagoya, Japan; 6 Department of Life Sciences and Health, Faculty of Health Sciences, Oslo Metropolitan University, Oslo, Norway; 7 Institute for Biomedicine and Glycomics, Griffith University, Gold Coast, Queensland, Australia; 8 School of Environment and Science, Griffith University, Gold Coast, Queensland, Australia; 9 Medical AI Center, Niigata University School of Medicine, Niigata, Japan; 10 Kavli Institute for Nanoscience Discovery, University of Oxford, Oxford, United Kingdom; 11 Complex Carbohydrate Research Center, University of Georgia, Geneva, Georgia, USA; 12 Chobanian & Avedisian School of Medicine, Department of Biochemistry & Cell Biology, Boston University, Boston, Massachusetts, USA; 13 Glycan and Life Systems Integration Center (GaLSIC), Soka University, Tokyo, Japan; 14 Projects, Symposia and PR, Beilstein-Institut, Frankfurt am Main, Germany

**Keywords:** MIRAGE, mass spectrometry, glycomics, glycoproteomics, MS-based identification, reporting guideline, glycoinformatics, FAIR, data standard

## Abstract

The MIRAGE (Minimum Information Required for A Glycomics Experiment) guidelines for mass spectrometry (MS) data were initially developed to standardize the reporting of instrumentation, data acquisition and analytical details of the MS-based identification of released glycans. However, the growing interest in the study of intact glycoproteins and recent advances in MS-based glycoproteomics now necessitate a revision and expansion of these guidelines. This update includes an enhanced section focused on glycan structure analysis (glycomics) and introduces a new component tailored to the specific requirements of glycoproteomics. It addresses both shared and unique aspects of each approach and highlights glycoinformatics resources designed to facilitate data submission in compliance with the updated standards

## Highlights

• MIRAGE reporting guidelines for the reporting of MS-based identification of glycans.
• First version widely adopted by journals and database maintainers. Growing interest in the study of intact glycoproteins and recent advances made a revision necessary.
• Update includes now components addressing the specific requirements of glycoproteomics data reporting.

## Graphical Abstract

**Figure:**
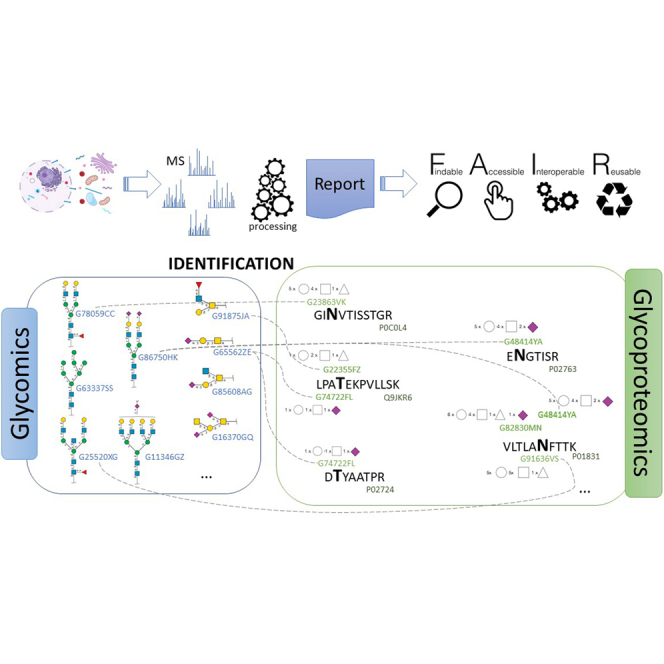

## In Brief

The primary aim of MIRAGE is to improve the quality of glycomics data in the scientific literature. Researchers seeking to understand the biochemical structure–function relationships of carbohydrates require detailed descriptions of the assay conditions and the experimental results. Often these data are insufficiently reported in the literature and thus, the working group develops reporting guidelines. Here, we propose guidelines for the reporting of mass spectrometry experiments for the identification of both released glycans and glycoconjugates, in particular glycoproteins.

## Background of Electronic Data Acquisition Infrastructure in Glycomics

Almost 20 years ago, a group of glycoscientists proposed a course of action for developing a worldwide linked electronic infrastructure for the deposition, registration, storage and analysis of carbohydrate structure data (1, 2). Such an endeavor was deemed essential to advance the field and would not only require a physical infrastructure, i.e. databases and software and to motivate the implementation of global public glyco databases such as UniCarb-DB (3) but also to define formal “standardized protocols,” including the definition of metadata, structural frameworks and data exchange formats. These protocols are commonly accepted and adopted, especially in the omics fields, and at least two recognized advantages can be observed. Firstly, comprehensive and standardized reporting of experimental data enables reproducibility. Secondly, the quality of published structural and/or functional data and their interpretation can be evaluated and the deposited data can be re-interrogated to address other research questions. Eagerness to implement the blueprint defined in (1) led to the foundation of the MIRAGE (Minimum Information Required for A Glycomics Experiment) project in 2011 under the auspices of the Beilstein-Institut (www.beilstein-institut.de/en) with the aim of meeting infrastructure-building requirements (4). The first objective became the definition of guidelines for the publication of glycomics data using mass spectrometry.

Reporting the results of a glycomics experiment is particularly demanding due to the molecular and biological complexity of glycosylation and the reality that the reported glycan structures are often incompletely resolved. For example, ambiguity may be created by indistinguishable masses of galactose, mannose, or glucose (all hexoses) or by non-assignable glycosidic bonds. Structural diversity, in the form of micro-heterogeneity (glycan distribution at each site) and macro-heterogeneity (glycan occupancy at each site), is another inherent feature of glycosylation that adds complexity to the analysis and reporting of glycans and glycoproteins. Even though experiments are geared to specify the identity and quantity of the complex carbohydrate structures in a sample, additional human expertise is still needed to refine molecular features and map the particular biosynthetic mechanisms. Thus, the comprehensive description of the techniques applied (instrumentation), the methods used (protocols) and the assumptions made for the subsequent analysis need to be defined to enable other scientists to understand, interpret and corroborate the findings or possibly need to re-analyze the data. Since mass spectrometry (MS) is the most widely used technique in glycomics and glycoproteomics, the MIRAGE Commission had initially developed guidelines for reporting mass spectrometry analysis data of free/released glycans (1). Moreover, repositories for depositing glycomics data, following these guidelines, were also developed in the form of UniCarb-DR (5) and GlycoPOST (6). UniCarb-DR was originally developed assuming that researchers would use the GlycoWorkbench software (7, 8) for annotating glycans in their mass spectra. However, GlycoWorkbench does not allow the import of raw MS data, and thus GlycoPOST became the repository for this type of data. Both repositories are available from the GlyCosmos Portal (9), a comprehensive resource for integrated glycan-related information. It includes GlyTouCan (10) and GlyComb (11), which assign accession numbers to glycans and glycoconjugates, respectively.

### The Need for Updated MIRAGE Mass Spectrometry Guidelines

The original MIRAGE guidelines for reporting Mass Spectrometry data (MS guidelines) attempted to provide an outline for the comprehensive description and metadata of the instrumentation, instrument setup and data acquisition protocols. However, expecting experimentalists to collect this wealth of information has been at the cost of limiting practical usage. As a result, the guidelines have not been widely adopted either by glycoscientists or by journals, thereby defying their stated purposes. This limited impact on improving glycomics data quality in the scientific literature led the MIRAGE Commission to release a tabular template to facilitate direct standardized and structured data input on a local computer (5). Furthermore, once filled, this table can be uploaded together with GlycoWorkbench files into the public GlycoPOST/UniCarb-DR repositories that were developed in parallel, for the purpose of collecting and centralizing standardized glycan data. This effort is consistent with other MS data management initiatives. GlycoPOST is a satellite repository of jPOST, a partner in the ProteomeXchange coordination body that manages and unifies the collection, storage, and access to proteomics data world-wide (12, 13). With these improved means of collecting and standardizing glycan structure information, the electronic data acquisition infrastructure for released/free glycans was established. However, the demand for infrastructure to support glycoproteomics, capturing both the glycan and protein information, has escalated with the increasing accessibility of MS instrumentation to biochemical laboratories and consequent rapid uptake in both the glycoanalytical and proteomic communities (14). The large-scale identification and quantitation of intact glycopeptides is still analytically challenging, and best practices are still evolving (15).

The current objective of the MIRAGE Commission is therefore to issue a set of standardized reporting guidelines to improve the data quality in glycoproteomics reporting.

## Other MIRAGE Reporting Guidelines

In addition to the MS guidelines, the MIRAGE Commission has developed guidelines for the reporting of:

• Glycan interaction analysis data (glycan and lectin arrays) (16, 17)
• Structural analysis data ((HPLC, NMR, CE) (18, 19)
• Sample preparation (20).

Note that the sample preparation guidelines (20) are considered as a common basis for any further MIRAGE reporting guidelines aimed at keeping the requirements for data analysis short and consistent. The entire list of MIRAGE guidelines is accessible at the MIRAGE project website (https://www.beilstein-institut.de/en/projects/mirage/guidelines/). In addition, all MIRAGE reporting guidelines are registered with FAIRSharing.org. A fruitful consequence of this joint effort in structuring data and metadata has been the rise of “spin-off” projects that include repositories cited above as well as others such as CarbArrayART (21) and GRITS (22).

The present article introduces an update of the MS guidelines for glycomics, including both an enhanced section dedicated to glycan structure analysis and a new component tailored to the specific requirements of glycoproteomics. It addresses both shared and unique aspects of each approach and highlights glycoinformatics solutions designed to facilitate data submission in compliance with the updated standards.

## A Combination of Guidelines to Support Mass Spectrometric Glycomics, Including Glycoproteomics

**Fig. 1:**
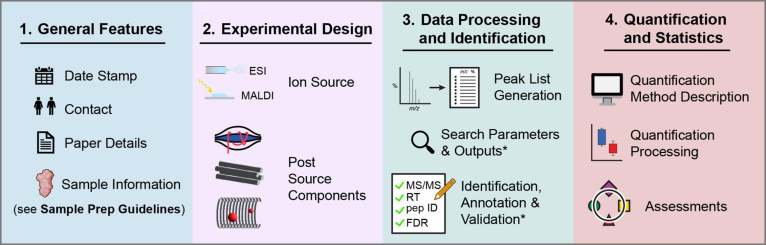
**The four major headings of MS data processing and reporting reflect the structure of the updated MS glycomics guidelines**.

Since the first MS guidelines for glycomics were published more than 10 years ago, high-throughput glycan structure identification using MS has expanded into the field of proteomics. In “glycoproteomics”, one attempts not only to identify sites in a protein chain on which a particular glycan is attached but also to unravel the site-specific glycan structures. While the first glycomics MS guidelines were quite generic and had a released glycan structure focus, the glycoproteomic methodology was not covered, mainly because technology was, at the time, underdeveloped. Although glycomics and glycoproteomics differ in their sample preparation methodologies, labeling, data searching/identification and quantitation, glycoproteomics shares many of the methods, data acquisition resources and infrastructure with proteomics. As a result, we have expanded the MIRAGE MS guidelines to cover glycomics and glycoproteomics while at the same time recognizing their differences in search result reporting. In addition, the quantitative aspects of the guidelines have been updated due to an evolution from structural to also include quantitative aspects (15). This updated version of the guidelines notes if a particular reporting item is required for either glycomics or glycoproteomics or both. The MIRAGE sample preparation guidelines, published separately (20), cover the reporting of sample preparation methods for both glycomics and glycoproteomics. The workflow is summarized in Figure 1.

## Updated MIRAGE MS Glycomics Guidelines

As mentioned in the introduction, the minimum reporting guidelines for glycomics MS were the first to be established in the MIRAGE family in 2013 (1) and subsequently were used as a starting point by the *Molecular & Cellular Proteomics* journal to generate the journal’s specific checklist for glycomics mass spectrometry data (https://www.mcponline.org/glycomic). Following this, MIRAGE liquid chromatography (LC) guidelines were published in 2019 (18). Observing that GlycoWorkbench remains the most popular open-source software assisting glycoscientists in identifying glycan structures from MS data (8), a readily MIRAGE-compliant tabular format was suggested for submission together with GlycoWorkbench files in order to capture structural information metadata.

Regarding glycoproteomics, the update is needed to account for the diverse sets of experimental approaches and data analysis strategies used across the field, along with the analytical challenges arising from profiling either *N*- or *O*-linked glycopeptides, or both (23). The current lack of maturity in glycoproteomics data analysis was illustrated in the first community study of the Human Glycoproteomics Initiative conducted under the Human Proteome Organization (HUPO) (24) and corroborated by other multi-laboratory comparison studies (e.g., (25)).

Consequently, the updated MIRAGE MS glycomics guidelines are designed to cover an extensive selection of biochemical methods, technological options, software tools, and computational methods as well as idiosyncrasies of glycoproteomic data collection and interpretation. The guidelines also feature recommended tools for specific reporting standards. The version proposed here has been drafted by members of the MIRAGE Commission with valuable community feedback from glycoproteomics practitioners Table 1.

**Table 1:** Overview of new MS Guidelines

1. General Features 2. Experimental Design 3. Data Processing & Identification a. Data Pointers b. Peak List Generation c. Glycomic & Glycoproteomic Search d. Glycoproteomic Search Output e. Peak List & Identification Annotation f. Identification Validation 4. Quantification & Statistics a. Quantification Method Description b. Quantification Processing c. Assessments

The guidelines are divided into four main sections with discrete subsections in the latter two. The General Features section contains information related to the publishing and upkeep of the data. The Experimental Design section focuses on summarizing sample information as the wet-lab experimental procedures are best captured through other guidelines. The Data Processing & Identification section focuses on the parameters affecting glycan and glycopeptide identification procedures, and the Quantification & Statistics section encompasses quantitation and analysis thereof.

The *Data Processing & Identification section* represents the bulk of these guidelines, aiming to record the nuances of the individual bioinformatics steps underpinning the large-scale glycan and glycopeptide identification process. This section encompasses end-to-end bioinformatics analysis reporting - from the raw data handling and transformation (if any) to the identification procedures and data storage. The first subsection *-Data Pointers-*focuses on data location details. In the second subsection, called *-Peak List Generation-*, the guidelines recommend reporting the details and parameters related to the software used for peak list generation from the raw files. The third subsection *-Glycomic & Glycoproteomic Search Parameters-*aims to encompass details of the identification software and strategies used, the glycan and protein target databases, and other factors relating to glycomic and glycoproteomic identification, including the reporting of identifications, which now includes usage of GlyTouCan glycan accession numbers; given the expansion of high-throughput glycoproteomics, proteomics parameters are also now included. The *Glycoproteomic Search Output reporting-*subsection encompasses protein-related details and recommends the usage of UniProt protein accession numbers in identification reporting. The fifth subsection *-Peak List & Identification Annotation-*encompasses evidence supporting the identifications and how identifications should be individually reported. The final subsection *-Identification Validation-*includes recommendations for reporting the manual validation of identifications.

The *Quantification & Statistics section* captures the details of the quantitation methodology and how data were statistically compared, including data normalization, standardization, aggregation, variance, and the types of computational methods used to assess the glycomics and/or glycoproteomics data. The first subsection *-Quantification Method Description-*revolves around reporting software details and parameters. The second subsection *-Quantification Processing*-focuses on transformations applied to raw quantitation data such as normalization, standardization measures, and aggregation. The third subsection*—Assessments—*broadly encapsulates the reporting of statistical analyses and visualizations.

The entire guidelines are included in the Supplemental Data section.

## Integration of GlyTouCan, GlycoPOST and UniCarb-DR repositories

GlycoPOST and UniCarb-DR are data repositories for glycomics data, publicly available in the GlyCosmos portal (www.glycosmos.org). Both have adopted the MIRAGE sample preparation and MS guidelines for glycan structures, but with different characteristics, as they have been developed and maintained separately by different groups, with limited cross-linking between their submissions. GlycoPOST is oriented toward hosting experimental glycan data, including raw mass spectrometric data, and supports fast data upload in a range of formats. One of the features of GlycoPOST is that authors can freely set the publication date of their submitted data and restrict access only to reviewers during the peer review process (i.e., the embargo period) and release the submitted data after the paper has been published. In parallel, UniCarb-DR is set up to capture not only overall experimental conditions, but also MIRAGE-compliant glycan structural assignment parameters through the recognition of GlycoWorkbench MS-based identification results. By virtue of this feature, the results of MS experiments can be displayed and searched in a web browser. Submitted content can be queried by a wide range of criteria, such as experimental metadata, species name, and/or monosaccharide composition data. While raw MS files upload is not supported, links to public MS repositories such as GlycoPOST or ProteomeXchange may be included.

From the submitter's point of view, it may be cumbersome to split the registration of identified glycans and the submission of raw data and identification results from the same MS experiment to two distinct repositories. This situation called for the integration of the two corresponding glycomics data repositories to improve and facilitate the submission process. This was recently achieved (6); the newly developed collaborative functions across GlycoPOST, GlyTouCan, and UniCarb-DR are briefly introduced below.

### Integration of the data submission workflow

Submitters can now submit all experimental data, including GlycoWorkbench files, only to GlycoPOST. This entails the removal of the data submission function in UniCarb-DR so that GlycoWorkbench files found in published projects on GlycoPOST will be periodically retrieved in a batch process and automatically registered in UniCarb-DR. This set-up allows UniCarb-DR users to set an embargo period via GlycoPOST as described above.

### MIRAGE LC guidelines *support* in GlycoPOST

UniCarb-DR previously accepted LC data before the MIRAGE LC guidelines were published. The current version of GlycoPOST has now been updated to accept MIRAGE-compliant LC data and subsequently outdated the UniCarb-DR LC submission, which has been removed from UniCarb-DR. Support for MIRAGE-compliant LC experimental data submission was added to GlycoPOST.

### Automatic registration of new glycan structures in GlycoWorkbench files into GlyTouCan

GlycoWorkbench files contain glycan structures annotated in their own annotation style format (.gwa). An application programming interface (API) was implemented to convert glycan data from.gwa into a format supported by GlyTouCan. It is now possible to automatically register glycans in GlycoWorkbench files into GlyTouCan to obtain GlyTouCan IDs, which can then be used to annotate the data in both GlycoPOST and UniCarb-DR.

As a result of these implementations, users now only need to register an account with GlycoPOST, and once they submit GlycoWorkbench files and make the GlycoPOST submission public, the glycomics data will be automatically registered into UniCarb-DR to take advantage of the added functionality the latter provides. For example, the mass spectra can be visualized together with the glycan fragments, and UniCarb-DR glycan entries will be linked to GlyTouCan.

In the end, the required parameters of the current MIRAGE MS guidelines for glycan structures can only partially be enforced in GlycoPOST and UniCarb-DR, mainly because neither of these platforms has fully implemented all these parameters into their data models. We are planning to continuously extend the services to enable researchers to submit their data in a MIRAGE-compliant way, which supports the reproducibility of the experimental results. For example, provision for obtaining glycopeptide unique identifiers was made through the recent release of a dedicated glycopeptide repository named GlyComb (11). Note that with these platforms, it is neither intended to produce standard operating procedures that specify how certain techniques should be applied nor attempt to provide benchmarks for the assessment of the data quality.

## Collaboration with Existing Standard Initiatives

It has always been part of the MIRAGE Commission's philosophy to consult the wider scientific community by drawing on external expertise to make the guidelines as precise and detailed as possible. Enabled by the Human Glycoproteomics Initiative under the Human Proteome Organization (https://hupo.org/Human-Glycoproteomics-Initiative), resulted in the development of some MS guidelines for the reporting of large-scale glycopeptide data. Glycopeptide identification faces additional complications, as there are many divergent identification methods and software tools. In an iterative process with members of HUPO-PSI, the guidelines were validated using real data with regard to both the sufficiency of the description of materials, methods and experimental data and the applicability of the guidelines in terms of understanding of the requested parameters. Bridging both omics disciplines, i.e., glycomics and proteomics, demonstrates that joint forces can avoid duplicate work.

Another successful step has been recently made with the extension of HUPO-PSI mzIdentML data exchange format to glycan-specific information. This format already solves most of the peptide- and protein-identification use-cases, but it also links experimental data and database files to the identification results. Its recent 1.3.0 (26) extension for crosslinking includes support for features shared with glycopeptide-like identifications based upon multiple spectra, and an update for glycoproteomics has been drafted. The core features in this proposal include support for referencing glycan structures and compositions by glycan accession number (GlyTouCan or GNOME (27)), or defined by controlled vocabulary terms. Additional guidance for how to report adducts, multi-glycosylated peptides, as well as ambiguous localizations common to collisional dissociation, are also covered. Based on this successful collaboration between the MIRAGE Commission and HUPO-PSI, a working group called PSI Glyco has been established within the organizational structure of HUPO-PSI (https://www.psidev.info/groups/mirage-glycosylation-working-group). This working group aims at collaborating with other HUPO-PSI working groups in the extension of existing data exchange formats and the development of new formats by joining forces to get the standard protocols accepted by researchers, journals and funding bodies. A common set of standard protocols will be adopted by the integration of the specific needs of both omics disciplines, but nothing should be developed from scratch again. In addition, cross-disciplinary expertise can be used to address the definition of a common terminology, ontology, and metadata.

## Discussion and Prospects

### FAIR Molecular Data

The expansion of FAIR (Findable Accessible Interoperable Reusable) principles observable across all omics disciplines (28), makes it essential to include the reporting of glycomics and glycoproteomics experiments in this landscape. Complying with standardization guidelines is the first step towards FAIRifying data. Such an approach creates a new momentum for easier finding, accessing and interrogating published data, and usage then gives important context that is often missing from studies published in current literature, allowing for greater interoperability and reusability of the relatively limited supply of glyco-data. Over time, this will build the basis for greater understanding and allow for the application of more sophisticated and informed computational methods.

The general shift toward data FAIRification in recent years has led funding agencies in many countries around the world to require a Data Management Plan (DMP) in most grant submission forms (29). The underlying motivation for establishing this plan is to raise the levels of accessibility and reproducibility of scientific studies. To reach this goal, researchers are therefore invited to channel data generated in a project into repositories from which data can be consulted or retrieved. This habit was very early taken on in several areas of life science. In fact, soon after the dissemination of the Sanger sequencing method, nucleotide sequences were collected in three main repositories (GenBank, EMBL, and DDBJ). Likewise, X-ray crystallography-based protein 3D structures were deposited in the Protein Data Bank. In both these examples, standard file formats and metadata collection have greatly helped the submission process. In contrast, the diversity of technologies and methods coexisting in proteomics and metabolomics has hindered a similar process in these fields that both heavily depend on mass spectrometry data. Nonetheless, these disciplines have initiated the process and achieved significant progress.

Lessons learnt from proteomics emphasize the need for coordinated action. There was a before and an after the implementation of ProteomeXchange (12) that transformed proteomics MS data submission, not only because of better-designed input interfaces but also due to the obvious dissemination potential offered by the multiplicity of submission sites. Taking stock of online activity in over 10 years (30) clearly demonstrates the validity of the approach. This sets a trend to follow in other MS-based omics, including glycomics. Moreover, oversights recognized by the proteomics community can be avoided, as for instance, the slow motion for collecting metadata in the earliest MS data repositories. Only very recently has this issue been addressed with the release of the Sample and Data Relationship Format (SDRF and for Proteomics: SDRF-Proteomics) in order to support the annotation of biological and technical metadata and to store the relationship between a sample and its respective data files (31). The release of updates to the MIRAGE guidelines and the proposed submission process presented in this article have benefited from this experience in proteomics.

The field of glycobiology is growing in both population and popularity, and it is critical to establish minimum reporting practices as early as possible. These updated MS guidelines for glycomics alleviate the dearth of reported information seen entering the public repositories; the earlier process of channeling information on MS-based glycan identification via published articles had limited success, probably due to the complexity of the requirements and for the absence of dedicated repositories at the time. This update is intended to simplify reporting, and it is tailored to suit submission into the described recently released repositories. In the same vein, these new guidelines are designed to meet the bare minimum reporting needs in an increasingly complex domain. In the end, the MIRAGE MS guidelines will enable better, more reproducible glycoscience.

### Toward a Tighter Knit of Omics Data

There are still many challenges ahead for analytical glycobiology, and these guidelines are designed as a minimum, not an end goal, with more work and collective efforts needed to ensure their impact is increased in the years to come. The guidelines themselves will continuously need revisions as mass spectrometry technologies and data analysis approaches change, and the glycoproteomics guidelines will require adaptation as proteomics reporting standards evolve and as other PTMs, such as acetylation, phosphorylation, and polysialylation, become more integrated into glycoproteomics analysis. Changes and expansions of related ontologies will have downstream effects, and the glycobiology community must remain aware of how the other -omics spheres are impacting it.

The MIRAGE guidelines were made by and in conjunction with key players in glycomics and glycoproteomics. For a successful, mutual benefit to the glycobiology community and interconnecting fields, the guidelines need to become an essential part of the glycoscience research workflow, and this requires the implementation of tools and templates that are straightforward and easy to use. They also need to have community buy-in, for those working in the field to not just see the collective benefit, but also the personal benefit of having data that is easy to locate and reassess. Repositories must also invest in this need, and those running them must understand that it is worth including these reporting standards into their architecture, to encourage and preserve their uptake. Another hurdle arises from the fact that glyco-data are stored in an array of different repositories that are not necessarily glycosylation focused, such as proteomics repositories. Future efforts need to include the integration of these data to ensure that this valuable information is not lost.

Finally, we point out that the meaning of “glycomics” is shifting toward reflecting the technology (mostly MS) used to identify and quantify both free glycans and all glycoconjugates, rather than just referring to analysis of free/released glycans from proteins. In an increasing number of publications, “glycomics” has become an umbrella term to encompass released glycans, glycoproteomics, glycolipidomics, GAGomics and more. Therefore, this manuscript describes MIRAGE MS guidelines for the glycomics umbrella term. The consequent implementation of software and databases spanning glycoconjugates matches this revised view.

## Supplemental data

This article contains supplemental data.

## Conflict of Interest

The authors declare that they do not have any conflicts of interest with the content of this article.
