# Supplementary material for: Update and New Implementation of the MIRAGE Reporting Guidelines for Mass Spectrometry Experiments in Glycoscience
Source: Mol Cell Proteomics. 2025 Nov 24;25(2):101473. doi: 10.1016/j.mcpro.2025.101473 (PMC12891849; doi:10.1016/j.mcpro.2025.101473)
Supplement: Supplementary Materials [file mmc1.docx]

| Section | Definition | Type of expected information | Helpful tools | Glyco-mics | Glyco-proteomics |
| --- | --- | --- | --- | --- | --- |
| 1. General Features | | | | | |
| Data stamp | The date on which the work described was completed | Standard date ‘YYYY-MM-DD’ format |  | X | X |
| Responsible person or role and contact information | The primary contact person’s name for this data set with affiliation and contact information | Short free text |  | X | X |
| Paper title and journal | The DOI of associated manuscript | URL (<https://doi.org>) |  | X | X |
| 1. Experimental Design | | | | | |
| No input required | See MIRAGE Sample Prepration Guidelines |  | [https://doi.org/10.3762/ mirage.1](https://doi.org/10.3762/mirage.1) | X | X |
| 1. Data Processing & Identification | | | | | |
| 3A. Data Pointers | | | | | |
| Location of raw data | If made available in a public repository, the corresponding URL, or the information on **how to retrieve the raw data**. If not made available for public access, name the contact person or source and the internal coordinates of the data. | URL data set identifier/password or short free textx | <http://www.proteomexchange.org>/  <https://glycopost.glycosmos.org/> | X | X |
| Location of processed data | If applicable, URL or the information on **how to retrieve processed data.** | URL, dataset  identifier/password or short  free text | <http://www.proteomexchange.org/>  <https://glycopost.glycosmos.org/> | X | X |
| Location of any associated data | If **complementary experiments** were conducted in association with the present experiment, pointers to corresponding standard-compliant metadata unless otherwise accounted for in this report. | URL, dataset  identifier/password or short  free text | <https://www.psidev.info/> | X | X |
| 3B. Peak List Generation | | | | | |
| MS raw data processing software (if applicable) | The **name of the software** and the **version number**.  Possibly specify commercial source or if open source.  If applicable, specify whether change(s) were made to the original program code that may affect the results. | PubMed ID, URL or URI, or short free text |  | X | X |
| MS raw data processing strategy | **Type of raw data processing** performed with the  software (e.g. de-isotoping, charge deconvolution, peak picking). If applicable, parameters for the  generation/selection/smoothing of peaks. | Short text with possible drop down list or PubMed ID, URL or URI | Draw terms from:  <https://tinyurl.com/EBIOLS>  if an mzML file is uploaded, these  can be extracted from it | X | X |
| Processed data file | Information about the processed MS data file. This includes the **file format and availability**; if applicable  the URI to access the file. Where available:  - the reference numbers of all the scans (as numbered in the raw file) that were combined to produce a peak list,  - the total number of acquisitions combined to produce  the peak list,  - whether the peak list was produced by summing or averaging the scans that are listed. | URI or short free text |  | X | X |
| 3C. Glycomic & Glycoproteomic Search | | | | | |
| Identification software  (if applicable) | The **name of the software** and the **version number**. Possibly specify commercial source or if open source.  If applicable, specify whether change(s) were made to the original program code that may affect the results. | PubMed ID, URL or URI, or short free text |  | X | X |
| Identification strategy | Search **strategy for precursor identification**, including if applicable, filtering of matches, use of database or spectral library, addition of manual pre- or post-search processing steps, inclusion of spiked synthetic glycans or glycopeptides, scoring methods for de-novo interpretation tools etc. | Short free text |  | X | X |
| Scoring algorithm | Method and software for **scoring** (for example, PSMs), along with the parameters supplied to the algorithm/method. | Short free text, and PubMed ID, or URL or URI |  | X | X |
| Taxonomy | **Taxonomic details** as an NCBI TaxId. If not applicable, name of species as known by submitter. | TaxID or short free text |  | X | X |
| Glycoconjugate search space | If database search, **type and version of the queried database(s)** and **glycan search space**.    If the database(s) is/are not available online, content details, including any information that will uniquely specify  molecules and enable access. | URL or short free text | <https://www.uniprot.org/>  <https://www.ncbi.nlm.nih.gov/refseq/> |  | X |
| Glycan database | List of **glycan compositions/structures** and/or the glycan masses considered in the search. Expected accession number in specified GlyTouCan version for each, unless none was assigned. | List of IDs | <https://glytoucan.org/>  <https://glycosmos.org/glycans/composition> | X |  |
| Spectral library | If spectral library search, type and size of spectral library (e.g., in-house or public) | Short free text |  | X | X |
| Specified protease cleavage | In silico **cleavage pattern** as available on the search engine as well as cleavage rules if they have been defined by the user. Note if the software cleavage rules differ from the experimental procedure | Short free text |  |  | X |
| Number of missed protease cleavages | Selected number of allowed **missed protease cleavage** sites | Number |  |  | X |
| Additional parameters related to protease cleavage | If applicable, details of **semi-specific protease cleavage(**s) | Short free text |  |  | X |
| Amino acid modifications | **Amino acid modifications** (other than glycans) and their mode (fixed or variable) considered in the search. When applicable, specify custom modification and associated rules. | Short text with possible drop down list |  |  | X |
| Glycan modifications | **Natural** or **induced glycan modification**, e.g., permethylation, acetylation, reduction. | Short text with possible drop down list |  | X | X |
| Allowed adducts | List of **adducts** that were allowed for the annotation. | List of masses |  | X | X |
| Loss and gain of small molecules | Allowed **loss and gain of small molecules**, e.g. -CH_2_ for undermethylation, loss of water or sialic acids | Short text with possible drop down list |  | X | X |
| Glycan fragmentation | List of allowed **glycan fragmentation** for the annotation run (A,B,C,X,Y,Z). This includes cross-ring and glycosidic cleavages. | Short text with possible drop down list |  | X |  |
| Precursor-ion and fragment-ion mass tolerance for tandem MS  (when applicable) | For MS1 and MSn queries, the **mass tolerance** of precursor and fragment ions permitted by the search engine. | Number |  | X | X |
| Thresholding and filtering | Other settings to the software that filtered out certain sequences from the database (e.g. allow only certain glycan types (e.g., N-glycan) or restriction by composition). This also includes the usage of threshold for scoring values. | Short free text |  | X | X |
| Any other relevant parameters | Any application-specific search parameters that may have an impact on the searched data and the interpretation of the results. | Short free text |  | X | X |
| 3D. Glycoproteomic Search Output | | | | | |
| Data file | Information about the software output data file. This includes the **file format and availability**; if applicable the URI to access the file. | URI or short free text |  | X | X |
| Identified proteins | Protein accession number (e.g. UniProt) of all reported glycopeptides | List of IDs | <https://www.uniprot.org/>  <https://www.ncbi.nlm.nih.gov/refseq/> |  | X |
| Peptide sequence | Peptide sequence of all reported glycopeptides (notify any deviation from the expected protein cleavage specificity). | List of peptide amino acid sequences |  |  | X |
| Peptide sequence modifications  (other than glycans) | List occurrence and position of amino acid modifications, whether artifactual (oxidized Met or Carbamidomethylated Cys) or natural (e.g, phosphorylation) or arising from amino acid variation(s). Refer to PTM-IDs when applicable. | List of positions of modified or variable amino acids | <http://www.unimod.org>  <https://www.uniprot.org/docs/ptmlist>  <https://bioportal.bioontology.org/ontologies/PSIMOD> |  | X |
| Glycosite position | Glycosylated amino acid positions in the source protein and not peptide sequence. For N-sites acknowledge the N-X-S/T motif. FASTA format is recommended due to potential positional shifts. | List of positions of glycosylated amino acids with corresponding reference to full protein sequence |  |  | X |
| 3E. Peak List & Identification Annotation | | | | | |
| The following lines have to be repeated for each identified feature in the mass spectrum. This data could be provided in a combined annotation file (e.g., GlycoWorkbench file). | For glycomics, the features below can be recorded in Glyco-Workbench, spectra can be uploaded, annotated and additional information can be uploaded in "other" for each spectra, including observed mass, scoring and validation parameters. |  |  | X |  |
| MS level | The **MS level** (e.g., MS2) at which spectra were acquired. | Short text with possible drop down list |  | X | X |
| Fragmentation type | Type(s) of **fragmentation** used to identify molecules e.g., HCD, EThcD, CID, HCD and EThcD | Short text with possible drop down list |  | X | X |
| Ion mode | The **ion mode** (positive or negative) | Selection between positive/negative |  | X | X |
| Retention time | The **retention time** of all reported detected glycomolecules (eg glycans, glycopeptides) (in minutes). | List of numbers |  | X | X |
| Precursor *m/z* and charge | For MS2 spectra only, the **precursor** *m/z* value and the **charge state** of the precursor ion. | List of numbers |  | X | X |
| Mass data | The observed and expected masses | List of numbers |  | X | X |
| Confidence level | The **confidence level** of all reported glycopeptides^(e.g., score or FDR). | List of numbers | <http://www.grits-toolbox.org/>  <https://glycoworkbench.software.informer.com/2.1/> |  | X |
| Glycans | Details regarding the **glycan type** (e.g., N-, O-...) and **composition** (e.g. Hex5HexNAc2), GlyTouCan  accession number. | List of numbers | <https://glycosmos.org/glycans/composition>  <https://glytoucan.org/>  <https://glyconnect.expasy.org/compozitor/> | X | X |
| Glycan structure features | The **glycan structural features** inferred from observed data (previous row. | Types: Composition, topology, fully defined |  | X | X |
| Orthogonal approaches | Additional information used for evaluation of confidence. This may include the exoglycosidase treatment, lectin recognition, reference/internal database/standard etc. (e.g., unique fragmentation or PGC-specific retention  time patterns,...). | Short text with possible drop down list |  | X |  |
| Any other relevant information | Any additional information that has an impact on identification. | Short free text |  | X | X |
| 3F. Validation | | | | | |
| Manual curation and validation | If applicable, **validation** of the output data and identifications (e.g., manually curated/filtered). | Short free text with possible drop down list |  | X | X |
| Structural ambiguity | Can the data explain the type of monosaccharides, sequence, linkage position and configuration and site position of the reported glycan on the peptide carrier? | Short free text with possible drop down list |  | X | X |
| 1. Quantification & Statistics | | | | | |
| 4A. Quantification Method Description | | | | | |
| Quantification method | Methodology used for quantification (e.g., MS1 precursor intensity, MS2 fragment intensity, duplex stable isotope labeling, multiplex isobaric tag labeling, label-free method based on spectral count, etc.); if a methods paper is cited, any significant deviations should be noted. | PubMed ID or short text with possible drop down list |  | X | X |
| Quantification software | The **name of the software** and the **version number**. Possibly specify commercial source or if open source. If applicable, specify whether change(s) were made to the original program code that may affect the results. | PubMed ID, URL or URI, or  short free text |  | X | X |
| Replicate information | The number of sample or biological replicates and the number of technical replicates. | Numbers |  | X | X |
| Reference points | The quantitation and **standards used**. If the quantitation was not label-free, the labels used and any randomization system used with the labeling. | Short text with possible drop down list |  | X | X |
| 4B. Quantification Processing | | | | | |
| Data normalization | Performed **data normalization** (e.g. logarithmized or square rooted abundance). Description of, if any, the TIC proportionate normalization of abundance values. Describe TMT normalisation if applicable. | Short text with possible drop down list |  | X | X |
| Relative abundance transformation | Possible data transformation into **relative abundance** (e.g. at each site or for each protein), if at all. | Short text with possible drop down list |  | X | X |
| Other transformation technique | If any transformation was applied, details of the filtered  and/or processed input intensity values. | Short free text |  | X | X |
| Replicate aggregation | The calculation method **aggregating / combining the values** from experimental replicates and/or groups (e.g., average, geometric average, weighting, etc. ...) | Short text with possible drop down list |  | X | X |
| Evaluation method | **Evaluation method** applied to the quantification software (or manual calculation) result. | Short text with possible drop down list |  | X | X |
| Acceptance criteria | The **acceptance criteria** and quantitative measures of variability (e.g. standard error). | Short text with possible drop down list |  | X | X |
| Data variance | If applicable, **observed variance** between technical replicates, within sample groups, and across the dataset. | List of numbers |  | X | X |
| Outliers | Definition of the range of observations and details of any outliers or exceptions in either samples or individual molecules. | Numbers and short free text |  | X | X |
| 4C. Assessments | | | | | |
| Data analysis software | The **name of the software** and the **version number**. Possibly specify commercial source or if open source. If applicable, specify whether change(s) were made to the original program code that may affect the results. | PubMed ID, URL or URI, or  short free text |  | X | X |
| Statistical test overview | **Statistical tests** performed and their parameters and thresholds of significance as applicable. If applicable, multiple testing corrections performed and reasoning behind the use of the applied tests. | Short text with possible drop down list |  | X | X |
| Clustering overview | Possible **clustering tests** performed, their parameters and thresholds of significance. If applicable, multiple testing corrections performed and reasoning behind the use of the applied tests. | Short free text |  | X | X |
|  |  |  |  |  |  |
